# Relationship between blood urea nitrogen to serum albumin ratio and short-term mortality among patients from the surgical intensive care unit: a population-based real-world study

**DOI:** 10.1186/s12871-023-02384-7

**Published:** 2023-12-19

**Authors:** Jinyu Zhang, Lei Zhong, Jie Min, Yunhai Wei, Lan Ding

**Affiliations:** 1 Department of Gastrointestinal Surgery, Huzhou Central Hospital, The Fifth School of Clinical Medicine of Zhejiang Chinese Medical University, Huzhou, 313000 China; 2 Department of Intensive Care Unit, Huzhou Central Hospital, The Fifth School of Clinical Medicine of Zhejiang Chinese Medical University, Huzhou, 313000 China; 3 Huzhou Central Hospital, Affiliated Central Hospital Huzhou University, Huzhou, 313000 China

**Keywords:** Blood urea nitrogen, Serum albumin, Surgical intensive care unit, Short-term mortality, MIMIC-IV database

## Abstract

**Background:**

Patients admitted to the surgical intensive care unit (SICU) often suffer from multi-organ dysfunction and have a high mortality rate. Therefore, finding a simple but effective clinical indicator to predict the prognosis of patients is essential to improve their survival. The aim of this study was to investigate the relationship between blood urea nitrogen to serum albumin ratio (B/A) and short-term mortality among patients from the SICU.

**Methods:**

All eligible adult patients admitted to the SICU from the Medical Information Mart for Intensive Care IV (MIMIC-IV) database were recruited for this study. Participants were divided into a death group (*n* = 638) and a survival group (*n* = 2,048) based on the 90-day prognosis, and then grouped by B/A quartiles. We used restricted cubic splines (RCS) to visually analyze the correlation of B/A with 30- and 90-day risk of death. Cumulative survival rates were estimated using Kaplan–Meier survival curves according to B/A quartiles and evaluated using the log-rank test. Cox proportional risk models were developed and sensitivity analyses were performed to explore whether B/A was independently associated with short-term outcomes in SICU patients. Receiver operating characteristic (ROC) curves were analyzed to ascertain the value of B/A for prognosticating 90-day outcome.

**Results:**

A total of 2686 participants were included in the final study, and their 30-day and 90-day all-cause mortality rates were 17.61% and 23.75%, respectively. The differences in 30-day and 90-day mortality rates were statistically significant among the four groups of patients (all *p* < 0.001). RCS curves showed that B/A was linearly associated with the risk of 30-day and 90-day all-cause mortality in SICU patients (χ2 = 0.960, *p* = 0.811; χ2 = 1.940, *p* = 0.584). Kaplan–Meier analysis showed that the 90-day cumulative survival rate gradually decreased as B/A increased, with patients in the highest quartile of B/A having the lowest survival rate (*p* < 0.001). Cox regression indicated that elevated B/A (> 9.69) was an independent risk factor for 30-day and 90-day all-cause mortality in SICU patients. The analysis of ROC curves demonstrated that B/A exhibited a significant predictive ability for 90-day mortality, with an optimal threshold of 6.587, a sensitivity of 56.9%, and a specificity of 64.8%.

**Conclusions:**

Elevated B/A (> 9.69) on admission was an independent risk factor for short-term mortality in SICU patients, and clinicians should pay more attention to this group of patients and intervene clinically at an early stage to reduce mortality.

**Supplementary Information:**

The online version contains supplementary material available at 10.1186/s12871-023-02384-7.

## Introduction

With the continuous progress of medicine and the construction of subspecialization, the surgical intensive care unit (SICU) has provided better protection for the treatment and care of surgical perioperative patients, especially for critically ill surgical patients, and thus has significantly reduced the mortality rate of patients [1–3]. Nevertheless, the number of patients admitted to SICU continues to increase, accounting for nearly one-third of ICU patients, and the mortality rate of SICU patients remains high, even up to 50% in some less developed countries [4–6]. Therefore, identifying high-risk factors for death in SICU patients are essential to reduce mortality.

Blood urea nitrogen (BUN) is a byproduct of protein breakdown within the human body and is primarily eliminated through renal excretion. Consequently, its concentration serves as a significant determinant of renal function, metabolic condition, and nutritional status [7]. A case–control investigation revealed that elevated BUN levels were associated with a heightened likelihood of postoperative stroke subsequent to cardiac surgery [8]. Additionally, serum albumin, a negative acute phase reactant, has demonstrated prognostic value in various critical ailments, indicating an unfavorable outcome [9, 10]. The blood urea nitrogen to serum albumin ratio (B/A) is a novel inflammation prognostic marker that is calculated from two simple indicators, including urea nitrogen and albumin. It has been reported that the B/A was an independent risk factor for the prognosis of patients with a variety of critical illnesses, including chronic heart failure [11], aspiration pneumonia [12], myocardial infarction [13], acute pulmonary embolism [14], sepsis [15], and gastrointestinal bleeding [16].

However, the aforementioned studies primarily concentrate on prognostic prediction in a singular critical illness, and there is a dearth of research investigating whether B/A also severs as a superior prognostic indicator for patients in the SICU. Consequently, this retrospective population-based study aims to examine the relationship between B/A and short-term outcomes (30-day and 90-day mortality) in patients admitted to SICU by gathering pertinent data from the Medical Information Mart for Intensive Care IV (MIMIC-IV) database.

## Methods

### Data source

Our study used data from the MIMIC-IV database (version 2.0), which contains data on 315,460 hospitalized patients between 2008 and 2019. An institutional review board from Massachusetts Institute of Technology and Beth Israel Deaconess Medical Center approved the creation of the database. Two of the authors completed the online training course of the National Institutes of Health to obtain approval for use of this database (Record ID: 51774135; 36142713). The data was anonymized to protect patient privacy.

### Study participants

Participants were selected for this study based on the following inclusion criteria: (1) Adult patients (age ≥ 18 years); (2) Patients admitted to the SICU for the first time. Subjects were excluded according to the following criteria: (1) Length of ICU stay < 24 h; (2) Died within 24 h of ICU admission; (3) Lack of key data such as BUN or albumin.

### Variable extraction

We extracted the following variables from the MIMIC-IV version 2.0 database: sex, age, laboratory parameters, comorbidities, Sequential Organ Failure Assessment (SOFA) score, Simplified Acute Physiology Score (SAPS) II, use of mechanical ventilation (MV), length of ICU stay and hospital stay. Laboratory parameters included BUN, albumin, white blood cell (WBC), hemoglobin, hematocrit, platelet, creatinine, bicarbonate, glucose, total calcium, phosphorus, magnesium, chlorine, potassium, and sodium. Comorbidities included hypertension, diabetes, acute respiratory failure (ARF), atrial fibrillation, cirrhosis, chronic kidney disease (CKD), malignancy, acute myocardial infarction (AMI), subarachnoid hemorrhage (SAH), acute pancreatitis, sepsis, peripheral vascular disease, severe liver disease, acute kidney injury (AKI), peptic ulcer disease, and paraplegia. The B/A ratio was calculated from the ratio of BUN (mg/dL) to albumin (g/dL). The SOFA score, SAPS II score, and all laboratory parameters were based on data collected within 24 h of admission to the SICU.

### Groups and outcomes

Participants were divided into a death group (*n* = 638) and a survival group (*n* = 2,048) based on the 90-day prognosis. The enrolled patients were grouped by B/A quartiles as follows: Q1, < 3.67 (*n* = 676); Q2, 3.67–5.52 (*n* = 667); Q3, 5.52–9.69 (*n* = 672); Q4, > 9.69 (*n* = 671). The primary outcome indicator for the study was 90-day (with SICU admission as the starting point) all-cause mortality, and the secondary outcome indicator was 30-day all-cause mortality.

### Statistical analysis

The basic clinical characteristics of patients were analyzed according to the death and survival groups. Data were expressed as mean and standard deviation or median (interquartile range) for continuous variables and number (percentage) for categorical variables. We compared categorical, normally and non-normally distributed continuous variables using chi-square test, t-test and Wilcoxon rank-sum test, respectively.

We used restricted cubic splines (RCS) to visually analyze the correlation of B/A with 30- and 90-day risk of death in SICU patients. Cumulative survival rates were estimated using Kaplan–Meier survival curves according to B/A quartiles and evaluated using the log-rank test.

We then used univariate and multivariate Cox regression models to estimate the relationship between B/A and short-term mortality in SICU patients. Variables with univariate *P* < 0.1 were included in the multivariate Cox regression analysis, and three models were finally constructed. Model I adjusted for nothing. Model II adjusted for age, SOFA score, WBC, hemoglobin, hematocrit, platelet, creatinine, bicarbonate, glucose, phosphorus, magnesium, chlorine, potassium. Model III, based on model I and model II, but further adjusted for MV, ARF, AMI, atrial fibrillation, SAH, cirrhosis, sepsis, CKD, malignancy, peripheral vascular disease, severe liver disease, AKI, paraplegia. Results were expressed as hazard ratio (HR) and 95% confidence interval (CI).

Receiver operating characteristic (ROC) curves were analyzed to ascertain the value of B/A for prognosticating 90-day outcome.

All analyzes were performed using Stata14.0 software and R language (version 4.2.0). A two-sided *P* value less than 0.05 was considered statistically significant.

### Sensitivity analysis

To further assess the robustness of the results, we performed several sensitivity analyses. First, considering that the ability of the liver to synthesize albumin would be affected, we excluded patients with cirrhosis and severe liver disease for sensitivity analyses, respectively. In addition, considering that human albumin infusion prior to ICU admission may have some effect on the B/A value, we excluded patients who had received human serum albumin infusion 48 h before ICU admission for sensitivity analysis.

## Results

### Study population and basic clinical characteristics

**Fig. 1:**
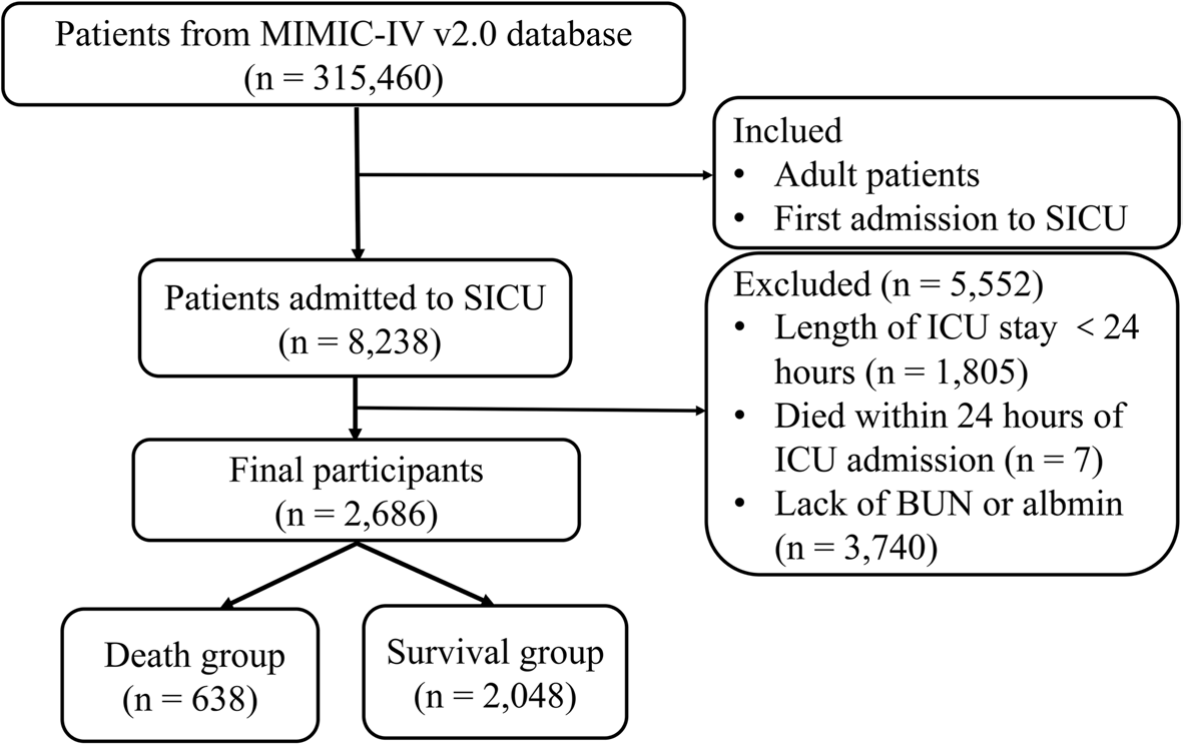
The inclusion and exclusion criteria of study participants

Overall, a total of 2686 participants were included in the final study according to the inclusion and exclusion criteria (Fig. 1). The basic clinical characteristics of all participants were shown in Table 1. The age of included patients was 63.13 ± 16.60 years, of which 1,489 (55.44%) were males and 1,197 (44.56%) were females. The main comorbidities were AKI (62.62%), sepsis (61.73%) and hypertension (43.86%). Patients in the death group were older and had higher SOFA scores, SAPS II scores, and higher proportions of mechanical ventilation. They had higher WBC, BUN, creatinine, phosphorus, magnesium, and potassium, and lower hemoglobin, hematocrit, platelet, and bicarbonate. These patients were also more likely to have ARF, atrial fibrillation, cirrhosis, CKD, malignancy, AMI, SAH, sepsis, peripheral vascular disease, severe liver disease, AKI, and paraplegia. The duration of hospital and ICU stays were shorter for patients in the death group.

**Table 1:** Basic clinical characteristics of the study population

Characteristic	Total (*n* = 2,686)	Death group (*n* = 638)	Survival group (*n* = 2,048)	t/Z/χ2 value	*P* value
Age (years)	63.13 ± 16.60	69.06 ± 15.61	61.29 ± 16.47	-10.528	< 0.001
Male, n (%)	1,489 (55.44)	343 (53.76)	1,146 (55.96)	0.949	0.330
SOFA (score)	5.93 ± 4.00	7.65 ± 4.41	5.39 ± 3.70	-12.834	< 0.001
SAPS II (score)	37.10 ± 14.44	45.42 ± 14.68	34.52 ± 13.35	-17.574	< 0.001
BUN (mg/dL)	18 (12,28)	23 (15,39)	17 (12,2)	-10.746	< 0.001
Albumin (g/dL)	3.20 ± 0.67	3.09 ± 0.70	3.23 ± 0.66	4.593	< 0.001
B/A	5.52 (3.67,9.69)	7.47 (4.58,13.46)	5.15 (3.41,8.41)	-10.798	< 0.001
WBC (× 109/L)	10.70 (7.50,14.80)	11.95 (8.80,16.70)	10.40 (7.30,14.20)	-6.655	< 0.001
Hemoglobin (g/L)	111.45 ± 22.71	107.57 ± 23.75	112.65 ± 22.24	4.958	< 0.001
Hematocrit (%)	33.42 ± 6.55	32.60 ± 6.95	33.68 ± 6.40	3.628	< 0.001
Platelet (× 10^9^/L)	192 (135,264)	185 (122,254)	194 (139,266)	2.204	0.028
Creatinine (umol/L)	79.56 (61.88,114.92)	97.24 (70.72,159.12)	79.56 (61.88,106.08)	-7.929	< 0.001
Bicarbonate (mmol/L)	22.68 ± 4.60	21.90 ± 5.22	22.92 ± 4.36	4.897	< 0.001
Glucose (mmol/L)	8.22 ± 3.81	8.46 ± 4.10	8.14 ± 3.72	-1.861	0.063
Total calcium (mmol/L)	2.09 ± 0.25	2.09 ± 0.23	2.09 ± 0.26	-0.005	0.996
Phosphorus (mmol/L)	1.19 ± 0.46	1.25 ± 0.53	1.17 ± 0.43	-3.673	< 0.001
Magnesium (mmol/L)	0.79 ± 0.16	0.81 ± 0.17	0.78 ± 0.16	-4.840	< 0.001
Chlorine (mmol/L)	103.81 ± 6.31	103.43 ± 7.20	103.93 ± 6.00	1.751	0.080
Potassium (mmol/L)	4.12 ± 0.77	4.24 ± 0.87	4.08 ± 0.74	-4.540	< 0.001
Sodium (mmol/L)	137.98 ± 5.27	137.95 ± 6.21	137.99 ± 4.95	0.165	0.869
MV, n (%)	1,828 (68.06)	513 (80.41)	1,315 (64.21)	58.714	< 0.001
Comorbidities, n (%)					
Hypertension	1,178 (43.86)	295 (46.24)	883 (43.12)	1.927	0.165
Diabetes	719 (26.77)	176 (27.59)	543 (26.51)	0.286	0.593
ARF	675 (25.13)	266 (41.69)	409 (19.97)	121.996	< 0.001
Atrial fibrillation	608 (22.64)	214 (33.54)	394 (19.24)	56.836	< 0.001
Cirrhosis	459 (17.09)	128 (20.06)	331 (16.16)	5.224	0.022
CKD	390 (14.52)	122 (19.12)	268 (13.09)	14.281	< 0.001
Malignancy	526 (19.58)	153 (23.98)	373 (18.21)	10.278	0.001
AMI	116 (4.32)	38 (5.96)	78 (3.81)	5.429	0.020
SAH	224 (8.34)	69 (10.82)	155 (7.57)	6.708	0.010
Acute pancreatitis	67 (2.49)	15 (2.35)	52 (2.54)	0.071	0.790
Sepsis	1,658 (61.73)	454 (71.16)	1,204 (58.79)	31.512	< 0.001
Peripheral vascular disease	236 (8.79)	76 (11.91)	160 (7.81)	10.202	< 0.001
Severe liver disease	403 (15.00)	124 (19.44)	279 (13.62)	12.888	< 0.001
AKI	1682 (62.62)	487 (76.33)	1195 (58.35)	67.206	< 0.001
Peptic ulcer disease	74 (2.76)	21 (3.29)	53 (2.59)	0.899	0.343
Paraplegia	328 (12.21)	106 (16.61)	222 (10.84)	15.131	< 0.001
Length of ICU stay (days)	3.62 (1.95,7.76)	5.04 (2.42,10.16)	3.17 (1.89,6.86)	-7.520	< 0.001
Length of hospital stay (days)	10.31 (6.13,18.58)	10.75 (4.96,18.79)	10.19 (6.52,18.54)	2.023	0.043

*SOFA* sequential organ failure assessment, *SAPS II* simplified acute physiology score II, *WBC* white blood cell, *BUN* blood urea nitrogen, *B/A* blood urea nitrogen to serum albumin ratio, *MV* mechanical ventilation, *ARF* acute respiratory failure, *CKD* chronic kidney disease, *AMI* acute myocardial infarction, *SAH* subarachnoid hemorrhage, *AKI* acute kidney injury, *ICU* intensive care unit

Furthermore, an examination was conducted on the admission characteristics of patients admitted to the SICU, as outlined in Table 2. Among the entire cohort of patients included in the study, those who were transferred from the emergency room to the SICU exhibited the highest mortality rate, amounting to 25.62%. Additionally, a distinction was observed between the groups of patients who experienced death and those who survived in relation to general surgery and transplantation (*p* < 0.05).

**Table 2:** Admission characteristics of patients admitted to the SICU

Variables	Total (*n* = 2,686)	Death group (*n* = 638)	Survival group (*n* = 2,048)	χ2 value	*P* value
**Source of admission, n (%)**					
Emergency room	1,534 (57.11)	393 (61.60)	1,141 (55.71)	42.368	< 0.001
Physician referral	468 (17.42)	61 (9.56)	407 (19.87)		
Transfer from hospital	551 (20.51)	159 (24.92)	392 (19.14)		
Others	133 (4.95)	25 (3.92)	108 (5.27)		
**Urgency cases, n (%)**					
Emergency cases	2,223 (82.76)	556 (87.15)	1,667 (81.40)	11.277	0.001
Elective cases	463 (17.24)	82 (12.85)	381 (18.60)		
**Type of surgery, n (%)**					
General surgery	500 (18.62)	73 (11.44)	427 (20.85)	28.418	< 0.001
Neurosurgery	427 (15.90)	88 (13.79)	339 (16.55)	2.771	0.096
Orthopedic surgery	60 (2.23)	14 (2.19)	46 (2.25)	0.006	0.938
Transplant surgery	230 (8.56)	4 (0.63)	226 (11.04)	67.306	< 0.001

*SICU* surgical intensive care unit

### Relationship between B/A and all-cause mortality

For the entire study population, the 30-day and 90-day all-cause mortality rates were 17.61% and 23.75%, respectively. As seen in Table 3, the differences in 30- and 90-day mortality rates were statistically significant among the four groups of patients (χ^2^ = 97.957, *p* < 0.001; χ^2^ = 116.310, *p* < 0.001).

**Table 3:** Comparison of all-cause mortality among different groups

Characteristics	B/A				χ2	*P*
	** < 3.67 (*n* = 676)**	**3.67–5.52 (*n* = 667)**	**5.52–9.69 (*n* = 672)**	** > 9.69 (*n* = 671)**		
**30-day mortality, n(%)**	60 (8.87)	106 (15.89)	112 (16.67)	195 (29.06)	97.957	< 0.001
**90-day mortality, n(%)**	90 (13.31)	138 (20.69)	157 (23.36)	253 (37.70)	116.310	< 0.001

*B/A* blood urea nitrogen to serum albumin ratio

**Fig. 2:**
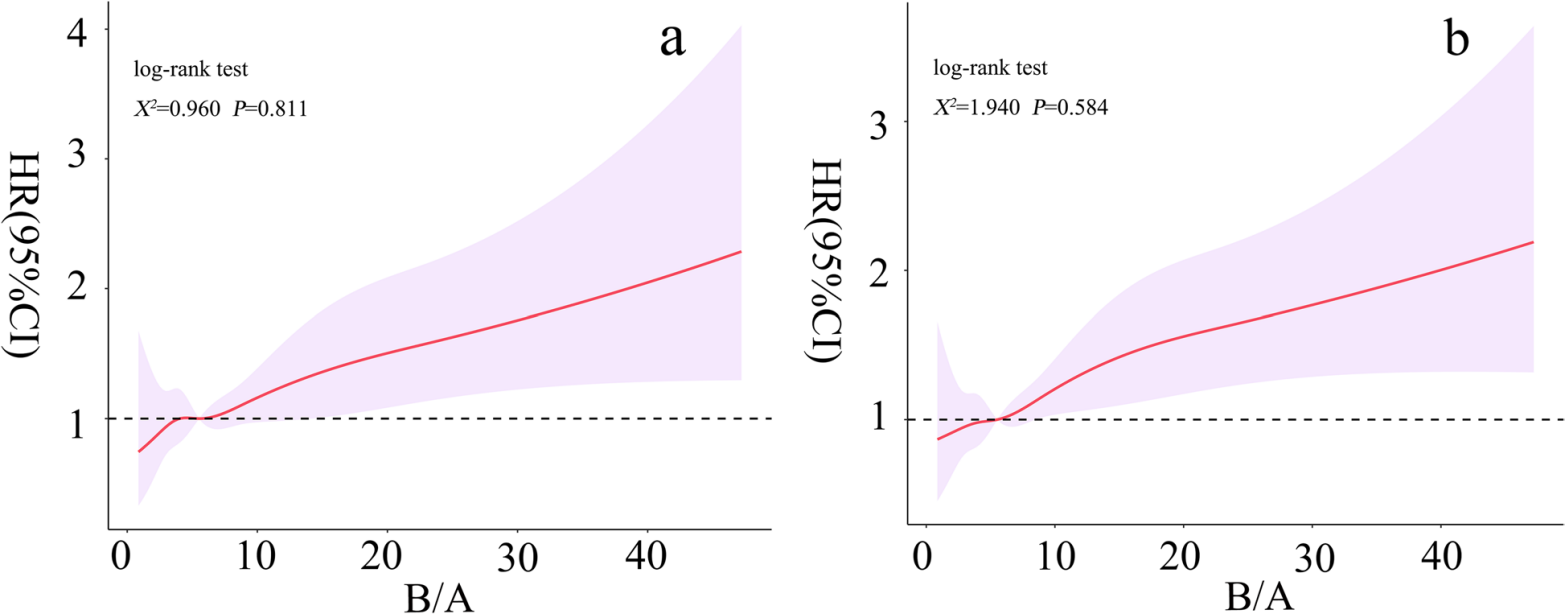
RCS curves of the relationship between B/A values and all-cause mortality risk in patients admitted to SICU

After adjusting for confounding variables, a linear association between B/A values and 90-day all-cause mortality risk in SICU patients was observed (χ2 = 1.940, *p* = 0.584), as indicated in Fig. 2b. In addition, a similar linear association was observed in the RCS curve of the relationship between B/A values and 30-day all-cause mortality risk (χ2 = 0.960, *p* = 0.811), as shown in Fig. 2a.

**Fig. 3:**
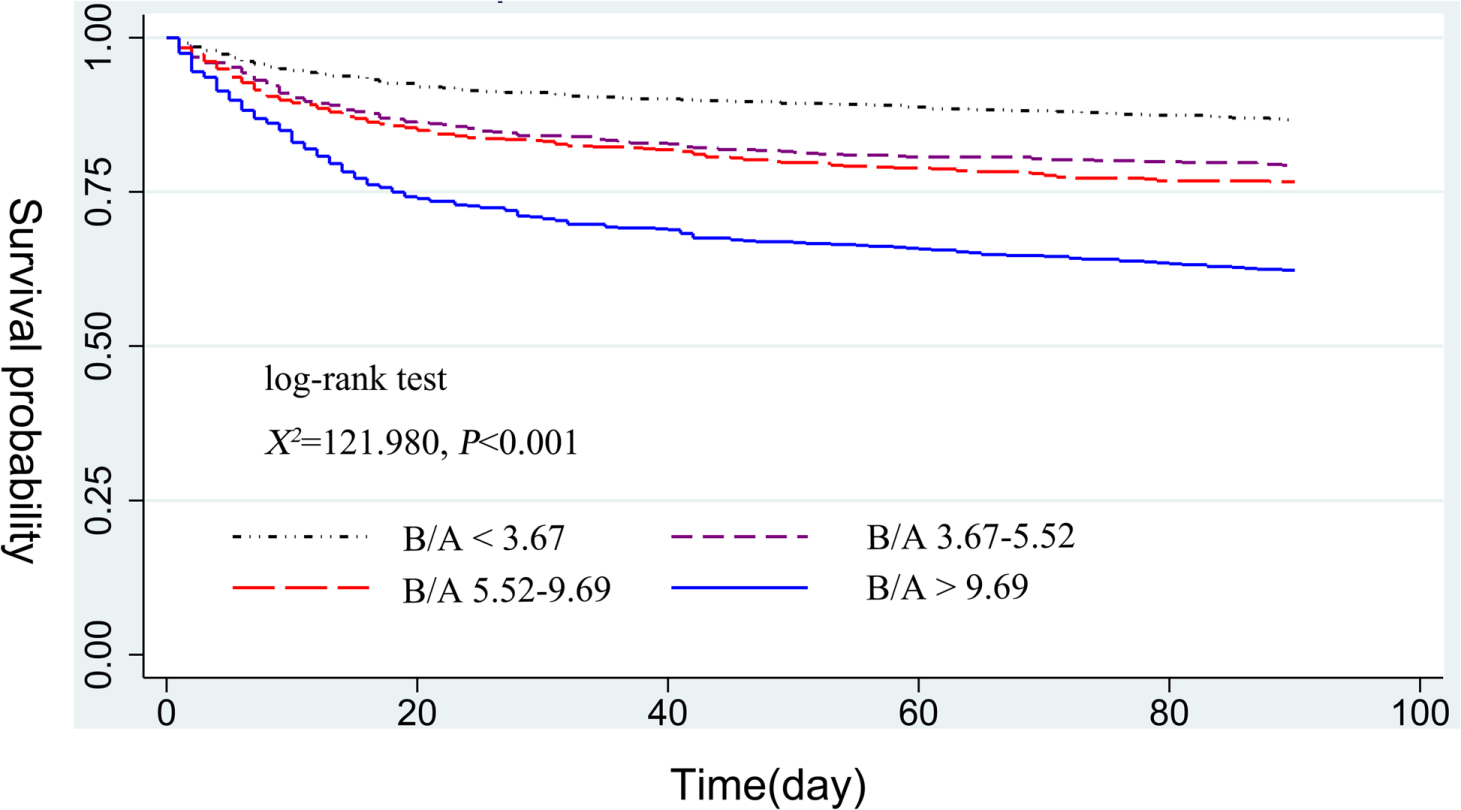
Kaplan–Meier curves of 90-day cumulative survival rates at various B/A values

Kaplan–Meier analysis in Fig. 3 comparing patients with different B/A values showed that the 90-day cumulative survival rate gradually decreased as B/A increased, with patients in the highest quartile of B/A having the lowest survival rate (log-rank test, χ2 = 121.980, *p* < 0.001). In addition, similar results were observed in the 30-day cumulative survival curves (Supplementary Fig. 1).

When analyzed as a continuous variable, B/A was associated with 90-day all-cause mortality. The HRs (95% CI) in the three models were 1.039 (1.032, 1.046), 1.019 (1.007, 1.030), and 1.021 (1.009, 1.033), respectively (all *p* < 0.001). When analyzed as quartiles in model I unadjusted for variables, the HRs (95% CI) for quartile 2, quartile 3 and quartile 4 were 1.633 (1.252, 2.129), 1.868 (1.442, 2.241) and 3.311 (2.602–4.211), respectively, compared with the reference group quartile 1 (all *p* < 0.001). Even after adjusting for a range of confounders, model III suggested that elevated B/A (> 9.69) was an independent risk factor for 90-day all-cause mortality in SICU patients (HR = 1.499, 95% CI = 1.100–2.041, *p* < 0.05). Similar results were observed in the analysis of 30-day mortality (Table 4).

**Table 4:** Multivariate Cox regression analysis of the association between different B/A levels and all-cause mortality

**Variable**	**Model I**			**Model II**			**Model III**		
	**HR**	**95%CI**	***P* value**	**HR**	**95%CI**	***P* value**	**HR**	**95%CI**	***P* value**
**30-day mortality**									
**B/A**	1.041	1.033–1.049	< 0.001	1.018	1.005–1.031	0.008	1.021	1.008–1.035	0.002
**Quintiles**									
**Q1 (< 3.67)**	Ref			Ref			Ref		
**Q2 (3.67–5.52)**	1.854	1.351–2.545	< 0.001	1.184	0.857–1.638	0.306	1.189	0.859–1.646	0.296
**Q3 (5.52–9.69)**	1.963	1.435–2.687	< 0.001	1.038	0.744–1.449	0.826	1.133	0.807–1.590	0.470
**Q4 (> 9.69)**	3.651	2.734–4.877	< 0.001	1.278	0.891–1.831	0.182	1.508	1.045–2.177	0.028
**p for trend**			< 0.001			0.322			0.032
**90-day mortality**									
**B/A**	1.039	1.032–1.046	< 0.001	1.019	1.007–1.030	0.001	1.021	1.009–1.033	< 0.001
**Quintiles**									
**Q1 (< 3.67)**	Ref			Ref			Ref		
**Q2 (3.67–5.52)**	1.633	1.252–2.129	< 0.001	1.102	0.840–1.447	0.483	1.086	0.827–1.427	0.553
**Q3 (5.52–9.69)**	1.868	1.442–2.241	< 0.001	1.059	0.802–1.396	0.687	1.104	0.833–1.463	0.493
**Q4 (> 9.69)**	3.311	2.602–4.211	< 0.001	1.329	0.982–1.799	0.066	1.499	1.100–2.041	0.010
**p for trend**			< 0.001			0.087			0.006

Model I adjusted for nothing

Model II adjusted for age, SOFA score, WBC, hemoglobin, hematocrit, platelet, creatinine, bicarbonate, glucose, phosphorus, magnesium, chlorine, potassium

Model III adjusted for model II plus MV, ARF, AMI, atrial fibrillation, SAH, cirrhosis, sepsis, CKD, malignancy, peripheral vascular disease, severe liver disease, AKI, paraplegia

*HR* hazard ratio, *95% CI* 95% confidence interval, *B/A* blood urea nitrogen to serum albumin ratio, *SOFA* sequential organ failure assessment, *WBC* white blood cell, *MV* mechanical ventilation, *ARF* acute respiratory failure, *AMI* acute myocardial infarction, *SAH* subarachnoid hemorrhage, *CKD* chronic kidney disease, *AKI* acute kidney injury

### Analysis of ROC curves

**Fig. 4:**
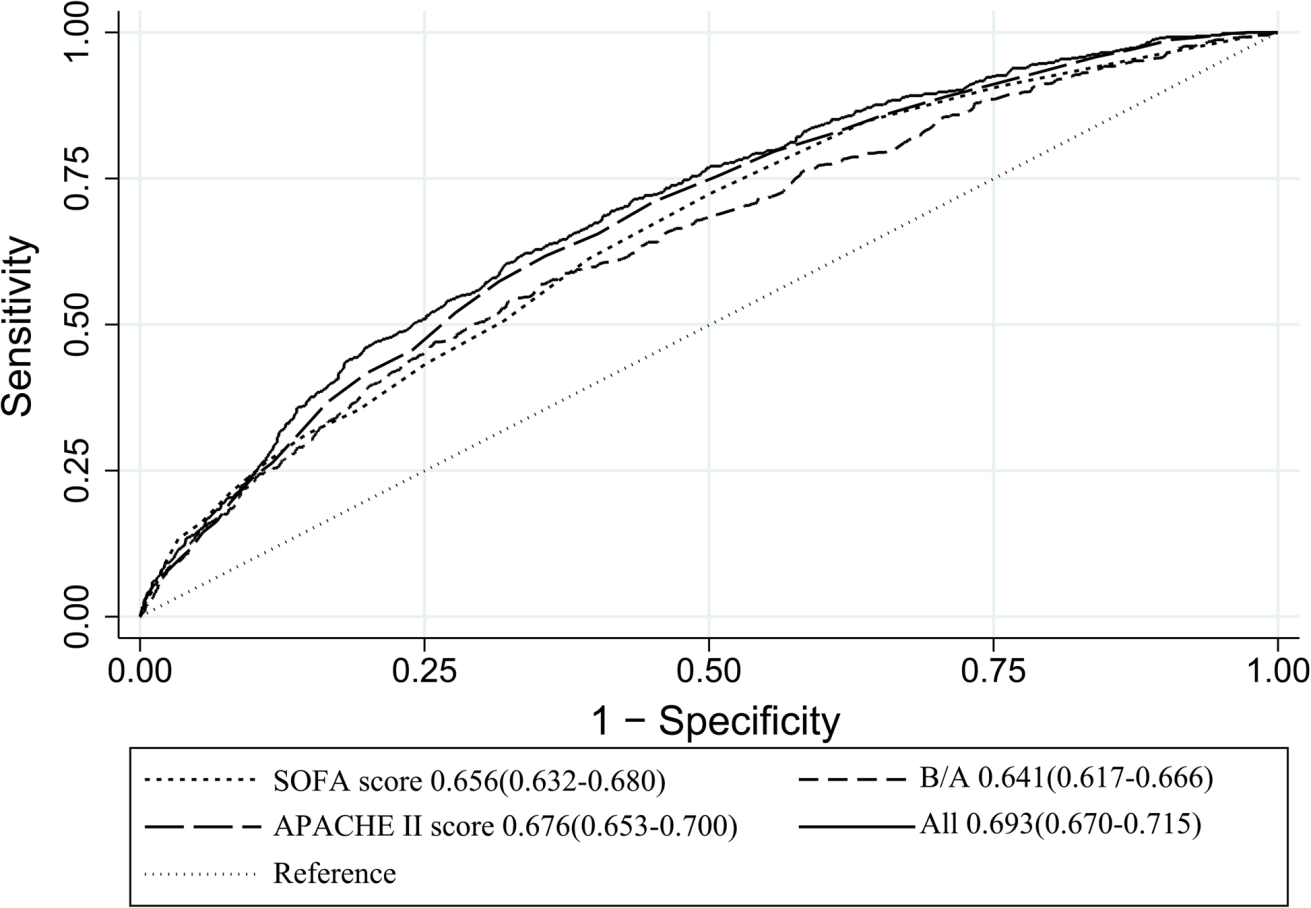
ROC curves for predicting 90-day mortality in patients admitted to SICU

The analysis of ROC curves demonstrated that B/A exhibited a significant predictive ability for 90-day mortality, with an optimal threshold of 6.587, a sensitivity of 56.9%, and a specificity of 64.8%. It is noteworthy that the predictive performance of B/A closely resembled that of the Acute Physiology and Chronic Health Assessment II (APACHE II) score and SOFA score (B/A area under the curve [AUC] = 0.641; APACHE II score AUC = 0.677; SOFA score AUC = 0.656). Moreover, the highest predictive performance was attained when B/A was combined with the APACHE II score and SOFA score (AUC = 0.693), yielding a sensitivity of 62.1% and specificity of 66.4% (Fig. 4 and Supplementary Table 1).

### Sensitivity analysis

After excluding 403 patients with severe liver disease, multivariate Cox regression analysis still suggested a significant association between elevated B/A levels and all-cause mortality among SICU patients (Supplementary Table 2). In addition, after excluding 112 patients who had received human serum albumin infusion 48 h before ICU admission, Cox regression analysis was again performed and the results were consistent with our main findings (Supplementary Table 3). Then, we again performed a sensitivity analysis after excluding 459 patients with cirrhosis and found that elevated B/A (> 9.69) remained an independent risk factor for 90-day all-cause mortality in SICU patients (Supplementary Table 4).

## Discussion

It is well-established that protein catabolism is heightened in critically ill patients, leading to an elevation in BUN levels. Renal reabsorption of urea nitrogen likewise affects the BUN levels of patients. Urea nitrogen is passively reabsorbed with water and sodium in the proximal renal tubule. In the more distal renal units, urea nitrogen reabsorption is also closely related to water reabsorption under the influence of antidiuretic hormone, which in turn is regulated by angiotensin II. When patients are dehydrated or have certain cardiovascular diseases occurring, sympathetic excitation and activation of the renin–angiotensin–aldosterone system can increase the reabsorption of urea nitrogen by the body. Therefore, high BUN level also reflects the state of renal hypoperfusion caused by hypovolemia or reduction of cardiac output [17]. One study has already reported that BUN levels at admission and at discharge were predictors of prognosis in patients with heart failure, and patients with high BUN levels had a poorer prognosis [18]. Additionally, serum albumin serves as a partial indicator of the organism's nutritional status and fulfills crucial functions such as maintaining plasma colloid osmolality, acting as an antioxidant, eliminating oxygen free radicals, serving as a carrier for various compounds, and inhibiting platelet activation and aggregation [19, 20]. Hypoalbuminemia has linked to decreased overall survival and increased recurrence rates in diverse malignant tumors [21, 22].

Notably, in recent studies, B/A has been employed as a novel prognostic predictor for critically ill patients. A study that included 800 hypertensive COVID-19 patients showed that urea nitrogen, albumin, and B/A were all valid predictors of in-hospital mortality, while B/A was a more reliable predictor than urea nitrogen and albumin [23]. Another study on the long-term prognosis of patients with acute myocardial infarction in the ICU found that a higher B/A (> 7.83) was associated with four-year mortality and was an independent risk factor for long-term mortality in such patients. In the ROC curve, the AUC value of B/A was higher than that of systemic inflammatory response syndrome (SIRS) score and APACHE II score [24]. In addition, there have been several studies showing that B/A has equally good predictive value in determining the prognosis of critically ill patients with certain surgical-related conditions. Allameh F et al. in their study analyzing the relationship between B/A and prognosis of patients with Fournier's Gangrene found that there was a significant difference between the B/A values of the death group and the survival group, and that elevated B/A was an independent predictor of death in patients with Fournier's Gangrene [25]. Ye L et al. collected data on 2527 cardiac surgery patients in the MIMIC database and analyzed factors associated with prognosis, and found a significant relationship between elevated B/A and hospital mortality. B/A might serve as an independent risk factor for adverse outcomes in patients undergoing cardiac surgery, and the investigators reached similar conclusions in an external validation cohort [26].

The present study, to the best of our knowledge, is the first to explore the association between B/A and the prognosis of patients admitted to the SICU. Our study found that patients in the death group were older and had higher SOFA scores, SAPS II scores, and higher proportions of mechanical ventilation. In this study, the enrolled patients were grouped by B/A quartiles and the differences in 30- and 90-day mortality rates were statistically significant among the four groups of patients. Kaplan–Meier analysis showed that the 30-day and 90-day cumulative survival rate gradually decreased as B/A increased, with patients in the highest quartile of B/A having the lowest survival rate. Cox regression analysis suggested that elevated B/A (> 9.69) was an independent risk factor for 90-day all-cause mortality in SICU patients. It is well known that albumin is produced by the liver, so when patients suffer from severe liver disease or cirrhosis, it can affect albumin levels [27, 28]. Therefore, this study conducted sensitivity analyses after excluding patients with severe liver disease or cirrhosis and those who had been infused with albumin within 48 h of admission to the hospital, and still ended up with the same conclusion.

The present clinical risk assessment of critically ill patients primarily relies on the utilization of APACHE II score, SOFA score, SAPS II score, and SIRS score [29, 30]. The prognostic predictive efficacy of these conventional scoring systems in the clinical management of critically ill patients is evident and has been validated in our study through the analysis of ROC curves. On this basis, researchers are actively engaged in the pursuit of novel and more straightforward prognostic markers. The analysis of ROC curves demonstrate that the AUC of the B/A ratio closely approximates the AUC of both the APACHE II score and SOFA score. Furthermore, when these three indices are combined, the AUC of the comprehensive index surpasses that of any individual index. This finding implies that B/A improves the prognostic predictive capacity of traditional scoring indicators, offering a novel viewpoint for clinicians and caregivers.

The current study has several advantages. First, the present study was innovative in exploring the correlation between B/A, a clinical indicator, and prognosis for SICU patients. Second, the study was more comprehensive in using 30-day and 90-day mortality to represent short-term prognosis. Finally, the sample size of this study was relatively large, and it is a large population-based real-world study.

However, this study still had some limitations. First, this study was a retrospective analysis with some bias. In addition, this study explored the correlation between B/A and short-term prognosis based on the initial value at the time of admission to the ICU, and did not further evaluate the correlation between B/A and long-term prognosis, and the dynamic changes of B/A levels. Finally, our analysis was limited to all-cause mortality and did not explore specific causes of death. Therefore, rigorously designed prospective studies are still needed to validate the findings of this study.

## Conclusion

To summarize, elevated B/A level (> 9.69) on admission was an independent risk factor for increased short-term mortality in SICU patients, and had some potential application as a clinically simple, inexpensive and easily accessible biomarker.

### Supplementary Information

**Additional file 1: Supplementary Figure 1.** Kaplan-Meier curves of 30-day cumulative survival rates at various B/A values.

** Additional file 2: Supplementary Table 1.** The prognostic capability of B/A, APACHE II score, SOFA score and the combined indicator for predicting 90-day all-cause mortality.

** Additional file 3: Supplementary Table 2.** Multivariate Cox regression analysis of the association between different B/A levels and all-cause mortality after excluding patients with severe liver disease.

** Additional file 4: Supplementary Table 3.** Multivariate Cox regression analysis of the association between different B/A levels and all-cause mortality after removing patients who had received human serum albumin infusion 48 hours before ICU admission.

** Additional file 5: Supplementary Table 4.** Multivariate Cox regression analysis of the association between different B/A levels and all-cause mortality after removing patients with cirrhosis.

## Data Availability

Publicly available datasets were analyzed in this study. This data can be found here: https://physionet.org/content/mimiciv/2.0/. The datasets used and/or analyzed during the current study are available from the corresponding author on reasonable request.

## Abbreviations

SICU: Surgical intensive care unit
BUN: Blood urea nitrogen
B/A: Blood urea nitrogen to serum albumin ratio
MIMIC-IV: Medical Information Mart for Intensive Care IV
SOFA: Sequential organ failure assessment
SAPS: Simplified acute physiology score
MV: Mechanical ventilation
WBC: White blood cell
ARF: Acute respiratory failure
CKD: Chronic kidney disease
AMI: Acute myocardial infarction
SAH: Subarachnoid hemorrhage
AKI: Acute kidney injury
RCS: Restricted cubic splines
HR: Hazard ratio
CI: Confidence interval
ROC: Receiver operating characteristic
AUC: Area under the curve
APACHE: Acute Physiology and Chronic Health Assessment
SIRS: Systemic inflammatory response syndrome

## References

[1] van Breugel J, Niemeyer M, Houwert RM, Groenwold R, Leenen L, van Wessem K (2020). Global changes in mortality rates in polytrauma patients admitted to the ICU-a systematic review. World J Emerg Surg.

[2] Zhang Y, Zhang J, Du Z, Ren Y, Nie J, Wu Z (2021). Risk factors for 28-day mortality in a surgical ICU: a retrospective analysis of 347 cases. Risk Manag Healthc Policy.

[3] Dhillon NK, Ko A, Smith E, Kharabi M, Castongia J, Nurok M (2017). Potentially avoidable surgical intensive care unit admissions and disposition delays. JAMA Surg.

[4] Vakayil V, Ingraham NE, Robbins AJ, Freese R, Northrop EF, Brunsvold ME (2020). Epidemiological trends of surgical admissions to the intensive care unit in the United States. J Trauma Acute Care Surg.

[5] Endeshaw AS, Fekede MS, Gesso AS, Aligaz EM, Aweke S (2022). Survival status and predictors of mortality among patients admitted to surgical intensive care units of Addis Ababa governmental hospitals, Ethiopia: a multicenter retrospective cohort study. Front Med (Lausanne).

[6] Aubry ST, Napolitano LM (2022). Management of common postoperative infections in the surgical intensive care unit. Infect Dis Clin North Am.

[7] Min J, Lu J, Zhong L, Yuan M, Xu Y (2022). The correlation study between blood urea nitrogen to serum albumin ratio and prognosis of patients with sepsis during hospitalization. BMC Anesthesiol.

[8] Arnan MK, Hsieh TC, Yeboah J, Bertoni AG, Burke GL, Bahrainwala Z (2015). Postoperative blood urea nitrogen is associated with stroke in cardiac surgical patients. Ann Thorac Surg.

[9] Jin X, Li J, Sun L, Zhang J, Gao Y, Li R (2022). Prognostic value of serum albumin level in critically Ill patients: observational data from large intensive care unit databases. Front Nutr.

[10] Eckart A, Struja T, Kutz A, Baumgartner A, Baumgartner T, Zurfluh S (2020). Relationship of nutritional status, inflammation, and serum albumin levels during acute illness: a prospective study. Am J Med.

[11] Lin Z, Zhao Y, Xiao L, Qi C, Chen Q, Li Y (2022). Blood urea nitrogen to serum albumin ratio as a new prognostic indicator in critical patients with chronic heart failure. ESC Heart Fail.

[12] Ryu S, Oh SK, Cho SU (2021). Utility of the blood urea nitrogen to serum albumin ratio as a prognostic factor of mortality in aspiration pneumonia patients. Am J Emerg Med.

[13] Balcik M, Satar S, Gulen M, Acehan S, Sevdimbas S, Acele A (2023). BUN/albumin ratio predicts short-term mortality better than SYNTAX score in ST-elevation myocardial infarction patients. J Cardiovasc Med (Hagerstown).

[14] Fang J, Xu B (2021). Blood urea nitrogen to serum albumin ratio independently predicts mortality in critically ill patients with acute pulmonary embolism. Clin Appl Thromb Hemost.

[15] Han T, Cheng T, Liao Y, Tang S, Liu B, He Y (2022). Analysis of the value of the blood urea nitrogen to albumin ratio as a predictor of mortality in patients with sepsis. J Inflamm Res.

[16] Bae SJ, Kim K, Yun SJ, Lee SH (2021). Predictive performance of blood urea nitrogen to serum albumin ratio in elderly patients with gastrointestinal bleeding. Am J Emerg Med.

[17] Sharma K, Mogensen KM, Robinson MK (2019). Pathophysiology of critical illness and role of nutrition. Nutr Clin Pract.

[18] Khoury J, Bahouth F, Stabholz Y, Elias A, Mashiach T, Aronson D (2019). Blood urea nitrogen variation upon admission and at discharge in patients with heart failure. ESC Heart Fail.

[19] Ronit A, Kirkegaard-Klitbo DM, Dohlmann TL, Lundgren J, Sabin CA, Phillips AN (2020). Plasma albumin and incident cardiovascular disease: results from the CGPS and an updated meta-analysis. Arterioscler Thromb Vasc Biol.

[20] Paar M, Fengler VH, Rosenberg DJ, Krebs A, Stauber RE, Oettl K (2021). Albumin in patients with liver disease shows an altered conformation. Commun Biol.

[21] Yamashita K, Ushiku H, Katada N, Hosoda K, Moriya H, Mieno H (2015). Reduced preoperative serum albumin and absence of peritoneal dissemination may be predictive factors for long-term survival with advanced gastric cancer with positive cytology test. Eur J Surg Oncol.

[22] Wu N, Chen G, Hu H, Pang L, Chen Z (2015). Low pretherapeutic serum albumin as a risk factor for poor outcome in esophageal squamous cell carcinomas. Nutr Cancer.

[23] Okşul M, Bilge Ö, Taştan E, Işık F, İnci Ü, Akın H (2023). Evaluation of the effect of bun/albumin ratio on in-hospital mortality in hypertensive COVID-19 patients. Eur Rev Med Pharmacol Sci.

[24] Zhao D, Liu Y, Chen S, Xu Z, Yang X, Shen H (2022). Predictive value of blood urea nitrogen to albumin ratio in long-term mortality in intensive care unit patients with acute myocardial infarction: a propensity score matching analysis. Int J Gen Med.

[25] Allameh F, Montazeri S, Shahabi V, Hojjati SA, Alinejad Khorram A, Razzaghi Z (2021). Assessment of the prognostic effect of blood urea nitrogen to serum albumin ratio in patients with Fournier’s gangrene in a referral center. Urol J.

[26] Ye L, Shi H, Wang X, Duan Q, Ge P, Shao Y (2022). Elevated blood urea nitrogen to serum albumin ratio is an adverse prognostic predictor for patients undergoing cardiac surgery. Front Cardiovasc Med.

[27] Bernardi M, Angeli P, Claria J, Moreau R, Gines P, Jalan R (2020). Albumin in decompensated cirrhosis: new concepts and perspectives. Gut.

[28] Arroyo V (2002). Review article: albumin in the treatment of liver diseases–new features of a classical treatment. Aliment Pharmacol Ther.

[29] Xu F, Li W, Zhang C, Cao R (2021). Performance of sequential organ failure assessment and simplified acute physiology score II for post-cardiac surgery patients in intensive care unit. Front Cardiovasc Med.

[30] Churpek MM, Snyder A, Han X, Sokol S, Pettit N, Howell MD (2017). Quick sepsis-related organ failure assessment, systemic inflammatory response syndrome, and early warning scores for detecting clinical deterioration in infected patients outside the intensive care unit. Am J Respir Crit Care Med.

