# Supplementary figures and images for: Relationship between blood urea nitrogen to serum albumin ratio and short-term mortality among patients from the surgical intensive care unit: a population-based real-world study
Source: BMC Anesthesiol. 2023 Dec 19;23:416. doi: 10.1186/s12871-023-02384-7 (PMC10729441; doi:10.1186/s12871-023-02384-7)

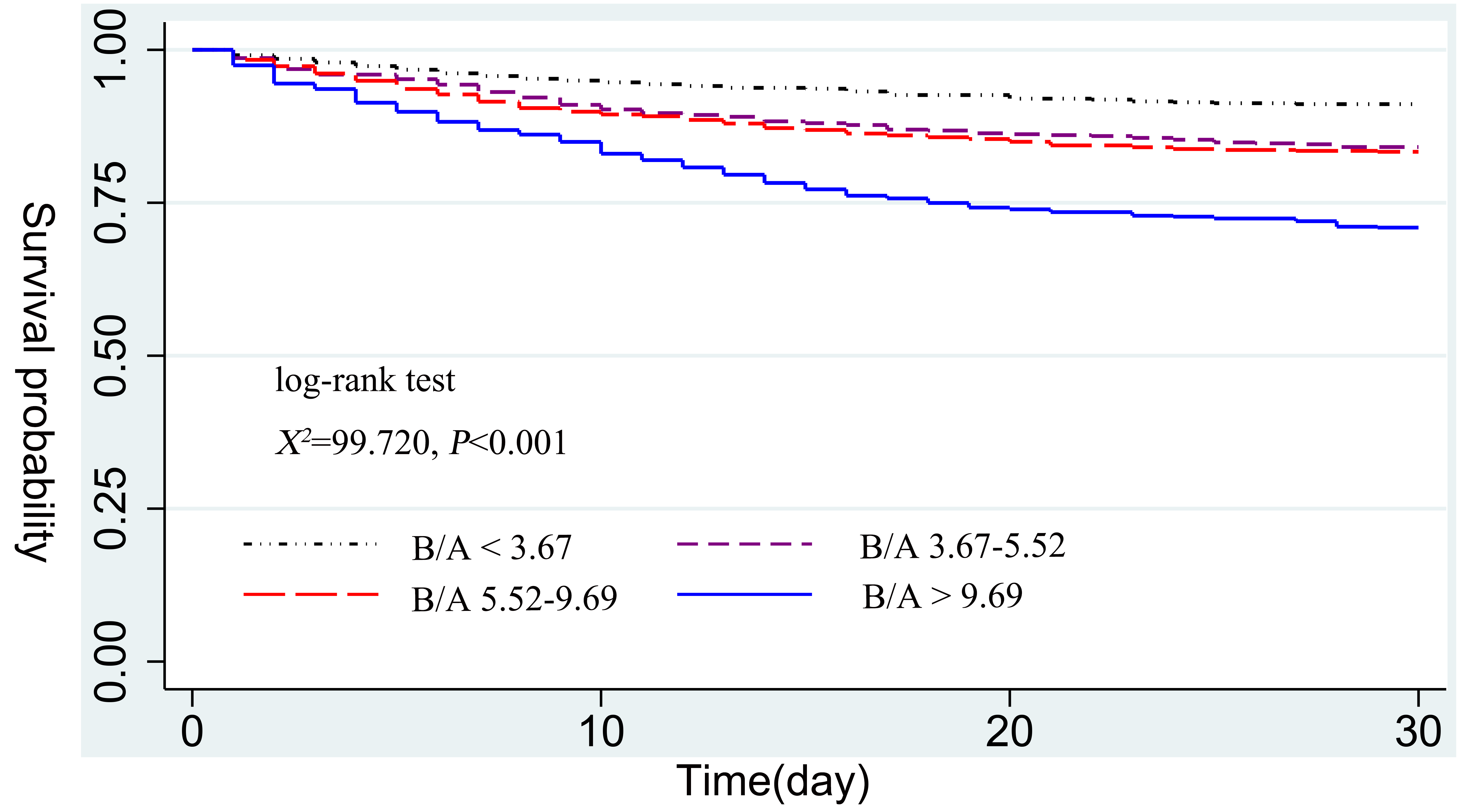

Supplement: Supplementary file 1 — Additional file 1: Supplementary Figure 1. Kaplan-Meier curves of 30-day cumulative survival rates at various B/A values. [file 12871_2023_2384_MOESM1_ESM.tif]
