# Supplementary material for: Relationship between blood urea nitrogen to serum albumin ratio and short-term mortality among patients from the surgical intensive care unit: a population-based real-world study
Source: BMC Anesthesiol. 2023 Dec 19;23:416. doi: 10.1186/s12871-023-02384-7 (PMC10729441; doi:10.1186/s12871-023-02384-7)
Supplement: Supplementary file 2 — Additional file 2: Supplementary Table 1. The prognostic capability of B/A, APACHE II score, SOFA score and the combined indicator for predicting 90-day all-cause mortality. [file 12871_2023_2384_MOESM2_ESM.docx]

**Supplementary Table 1** The prognostic capability of B/A, APACHE II score, SOFA score and the combined indicator for predicting 90-day all-cause mortality.

| Variables | threshold | AUC | 95%CI | sensitivity | specificity |
| --- | --- | --- | --- | --- | --- |
| B/A | 6.587 | 0.641 | 0.617-0.666 | 0.569 | 0.648 |
| APACHE II score | 20.500 | 0.676 | 0.653-0.700 | 0.618 | 0.644 |
| SOFA score | 4.500 | 0.656 | 0.632-0.680 | 0.724 | 0.499 |
| B/A+APACHE II score+SOFA score | 0.229 | 0.693 | 0.670-0.715 | 0.621 | 0.664 |

B/A, blood urea nitrogen to serum albumin ratio; APACHE II, acute physiology and chronic health evaluation II; SOFA, sequential organ failure assessment; AUC, area under the curve; CI, confidence interval.
