# Supplementary material for: Relationship between blood urea nitrogen to serum albumin ratio and short-term mortality among patients from the surgical intensive care unit: a population-based real-world study
Source: BMC Anesthesiol. 2023 Dec 19;23:416. doi: 10.1186/s12871-023-02384-7 (PMC10729441; doi:10.1186/s12871-023-02384-7)
Supplement: Supplementary file 5 — Additional file 5: Supplementary Table 4. Multivariate Cox regression analysis of the association between different B/A levels and all-cause mortality after removing patients with cirrhosis. [file 12871_2023_2384_MOESM5_ESM.docx]

**Supplementary Table 4** Multivariate Cox regression analysis of the association between different B/A levels and all-cause mortality after removing patients with cirrhosis.

| **Variable** |  | **model I** |  |  |  | **Model II** |  |  |  | **model III** |  |
| --- | --- | --- | --- | --- | --- | --- | --- | --- | --- | --- | --- |
|  | **HR** | **95%CI** | ***P* value** |  | **HR** | **95%CI** | ***P* value** |  | **HR** | **95%CI** | ***P* value** |
| **30-day mortality** |  |  |  |  |  |  |  |  |  |  |  |
| **B/A Quintiles** |  |  |  |  |  |  |  |  |  |  |  |
| **Q1 (<3.67)** | Ref |  |  |  | Ref |  |  |  | Ref |  |  |
| **Q2 (3.67-5.52)** | 2.142 | 1.529-3.000 | <0.001 |  | 1.363 | 0.966-1.925 | 0.078 |  | 1.326 | 0.939-1.873 | 0.109 |
| **Q3 (5.52-9.69)** | 2.224 | 1.583-3.125 | <0.001 |  | 1.163 | 0.809-1.670 | 0.415 |  | 1.249 | 0.866-1.803 | 0.235 |
| **Q4 (>9.69)** | 3.502 | 2.540-4.829 | <0.001 |  | 1.215 | 0.807-1.829 | 0.352 |  | 1.468 | 0.971-2.220 | 0.069 |
| **90-day mortality** |  |  |  |  |  |  |  |  |  |  |  |
| **B/A Quintiles** |  |  |  |  |  |  |  |  |  |  |  |
| **Q1 (<3.67)** | Ref |  |  |  | Ref |  |  |  | Ref |  |  |
| **Q2 (3.67-5.52)** | 1.843 | 1.393-2.439 | <0.001 |  | 1.229 | 0.922-1.637 | 0.160 |  | 1.177 | 0.883-1.569 | 0.265 |
| **Q3 (5.52-9.69)** | 2.019 | 1.525-2.671 | <0.001 |  | 1.109 | 0.821-1.497 | 0.501 |  | 1.147 | 0.846-1.553 | 0.378 |
| **Q4 (>9.69)** | 3.231 | 2.482-4.208 | <0.001 |  | 1.276 | 0.908-1.792 | 0.160 |  | 1.466 | 1.041-2.066 | 0.029 |

Model I adjusted for nothing.

Model II adjusted for age, SOFA score, WBC, hemoglobin, hematocrit, platelet, creatinine, bicarbonate, glucose, phosphorus, magnesium, chlorine, potassium.

Model III adjusted for model II plus MV, ARF, AMI, atrial fibrillation, SAH, sepsis, CKD, malignancy, peripheral vascular disease, severe liver disease, AKI, paraplegia.

HR, hazard ratio; 95% CI, 95% confidence interval; B/A, blood urea nitrogen to serum albumin ratio; SOFA, sequential organ failure assessment; WBC, white blood cell; MV, mechanical ventilation; ARF, acute respiratory failure; AMI, acute myocardial infarction; SAH, subarachnoid hemorrhage; CKD, chronic kidney disease; AKI, acute kidney injury.
